# Comparative analysis of media coverage concerning the social implications on three life sciences in Japan during 1991–2020

**DOI:** 10.3389/fsoc.2025.1523795

**Published:** 2025-03-11

**Authors:** Kohei F. Takeda, Megumi Komata, Kanako Takae, Mikihito Tanaka, Ryuma Shineha

**Affiliations:** ^1^Co-Design Center, Osaka University, Osaka, Japan; ^2^Graduate School of Interdisciplinary Science and Engineering in Health Systems, Okayama University, Okayama, Japan; ^3^Institute for Advanced Social Science, Waseda University, Tokyo, Japan; ^4^Faculty of Political Science and Economics, Waseda University, Tokyo, Japan; ^5^Research Center on Ethical, Legal and Social Issues, Osaka University, Osaka, Japan

**Keywords:** media discourses, media analysis, framing, biotechnology, science communication

## Abstract

Media coverage is an important determinant of the social conception and public understanding of science. Therefore, understanding the media framing of science and technology is important for science communication. As such, we try to determine the frames that are significant in news coverage concerning science and technology, whether the dominant frames changed over time, and whether there are any overlooked frames. To this end, we focused on news articles on multiple life-science fields in Japan to examine the ethical, legal, and social implications covered in the media of three fields: genetic modification, stem cell science and regenerative medicine, and brain-neuroscience. We examined seven frames (i.e., instrumental science, risky science, juggernaut science, techno-nationalism, governance, communication matters, and trust in science) related to the ethical and social implications for the three technologies. We collected 37,009 articles from the newspaper database. After a pilot analysis of the collected articles based on text mining, we coded a total of 1,805 articles from 1991 to 2020 using random sampling. Our results showed that the frames varied among the three technologies over time and no frame synchronization was observed. This implies that the media coverage of each technology was independent of those of the other technologies. A trend common to all technologies was that the frame “instrumental science” was dominant, meaning that positive opinions predominate in the Japanese media coverage of life sciences. This result suggests ethical issues of life sciences were often missing in Japanese media discourse. An urgent task is to bridge the gap between the discussions of ethics communities and the media coverage. Our study provides evidence of the potential social implications of life science according to assumed for public understanding.

## 1 Introduction

Modern life science has a significant impact on society and has been applied to many areas, from agriculture to medicine. The rapid progress of life science has not only many benefits, but also brings a variety of broad ethical, legal, and social implications to society. In the current knowledge-based society, it is essential to consider the various impacts and implications of life science.

Japan has had long-term public discussions on life science. After the 1990s, Japan experienced bovine spongiform encephalopathy (BSE) and genetically modified organism (GMO) controversies [mainly on the genetic modification (GM) of food], similar to European countries (Nishizawa, 2005; Shineha and Kato, 2009), which had various social implications. Previous studies pointed out the correlations between the media, public attitudes, and politics on such issues (Yamaguchi, 2013, 2020).

Regarding stem cell research and regenerative medicine, the Japanese government has financially supported induced pluripotent stem cells (iPSCs) since they were established by Japanese researchers in 2006. As such, there is hype and also high expectations regarding iPSCs and regenerative medicine in Japan (Shineha et al., 2010; Shineha, 2016; Shineha et al., 2018; Shineha et al., 2022). The Japanese government established a legal framework to promote regenerative medicine in 2014 (Konomi et al., 2015). However, as regenerative medicine by iPCSs has been promoted at the national level in Japan, Mikami, a sociologist, discussed the strength of an imaginary lock-in toward science policy based on a case study on stem cell research (Konomi et al., 2015). In 2015, a Japanese court ruled that a private clinic should pay compensation for damage to a patient who had received regenerative medicine therapies. This became the first worldwide reported case of reimbursement for regenerative medicine therapy for patient damage (Ikka et al., 2015).

Furthermore, the Japanese government has supported brain-neuroscience (BS) since the late 1980s. In the 2000s, the Japanese government initiated a funding program on BS and governmental bodies and academic societies published related guidelines (Gaillard, 2018). More recently, interdisciplinary research, particularly with AI, has been encouraged. As a result, new research projects on the social implications of BS have been conducted.

Scientific findings such as the ones described above relate to policymaking, hype, expectation, and concerns in both Japan and at the global level. Currently, the discussions considering the social aspects of science and society focus on responsible research and innovation (RRI). RRI has been regarded as a key concept in the discussions on science and technology policy as well (European Commission Horizon, 2020; OECD, 2022). The issues considered cover: broad public engagement in science and technology, increasing accessibility to scientific results, ensuring gender equality in both the research process and research content, encouraging of formal and informal science education, taking ethical, legal, and social implications (ELSI) (Stilgoe et al., 2013). According to Stilgoe and Guston, “Responsible innovation means taking care of the future through collective stewardship of science and innovation in the present” (Stilgoe and Guston, 2017, p. 1570). They also discussed the concept of RRI as having four key components: anticipation, inclusion, reflexivity, and responsiveness. In other words, RRI is a way of thinking about reflexive and adaptive innovation concerning emerging science and technology. This approach encourages us to develop legitimate and more effective processes of decision-making through open, transparent, and upstream dialog with various stakeholders, considering the previous experiences with GMOs, nanotechnologies, and others (Stilgoe et al., 2013; Stilgoe and Guston, 2017; Komiya et al., 2022).

As an important background of the discussions on RRI, we should mention “real-time technology assessment (RTTA)” (Guston and Sarewitz, 2002). Guston and Sarewitz discussed RTTA for better decision-making and communication between science and society and summarized the key components of RTTA as “research program mapping,” “analogical case study,” “communication and early warning,” and “technology assessment and choice” (Guston and Sarewitz, 2002). Concerning the key component of RTTA—“communication and early warning”—Guston and Sarewitz discussed how media analysis can function as an “early warning” to understand the social agenda behind emerging science (Guston and Sarewitz, 2002).

Regarding emerging life science, media coverage affects public perceptions, expectation, hype, and images of science through repeated contacts with discourses and framings (Shineha, 2016; Bauer and Gutteling, 2006; Lewison, 2007; Listerman, 2010; Marks et al., 2007; McCluskey et al., 2016; Nisbet and Huge, 2006; Nisbet and Lewenstein, 2002; Caulfield et al., 2016). Thus, understanding media framings on emerging science and technology is essential for understanding the related RRI and governance. However, few studies have comprehensively analyzed the media coverage on ELSI for multiple fields of life science. Thus, the use of media framings on ELSI for life science has not been examined in enough detail, which prevents a deep understanding of the relationship between press and politics (Nisbet and Huge, 2006; Nisbet and Lewenstein, 2002), as well as the background of the public discourses on life science.

Considering this gap, this study aims to identify the social implications embedded in Japanese newspaper articles about life science topics. This includes *what* types of problems are outlined with *what* kinds of assumptions and *how t*hey are described and contextualized with other societal issues. Thus, the research questions are as follows:

- What frames are significant in each field (regenerative medicine [RM], GM, and BS)?
- Have the dominant frames changed over time?
- Are there any overlooked frames in the news articles concerning life science?

## 2 Context of media analysis

In this study, we focus on newspaper articles. Newspapers are still the main source of discussion on new life science for the public in Japan, despite the advent of social networking. The use of news sources differs with age. The elderly use newspapers and television as primary information sources, while younger people use the Internet more. However, mass media remains a main information source for the public (Shineha et al., 2017). In addition, Internet news media often use newspapers and television as resources. McCombs and Valenzuela discussed that the agenda setting function of newspapers have been strengthened in the Internet era (McCombs and Valenzuela, 2020).

As an example of an analysis of Japanese media discourses on life science, Hibino and Nagata (2006, 2008) and Nagata et al. (2006) analyzed news articles in *Asahi-shimbun* (one of the major newspapers in Japan) from 1985 to 2004. Through content and correspondence analyses, they found drastic changes in the relationship between themes (e.g., biomedical use, agricultural use, genetic research), frames (e.g., economic prospects, ethics, pandora’s box, public accountability, globalization), actors, and so on. Meanwhile, the co-occurrences among the framings of “medical research,” “economy,” and “generic research” were stable and there were unique framings of emotional attachment toward cloned animals. Shineha et al. (2008) focused on news articles regarding GMOs from 1984 to 2006. Employing text mining, they found two shifts in the dominant themes. The first shift occurred in the late 1990s, from “medical and industrial application” to “GM food,” and simultaneously, the basic tone of the articles also shifted from positive to negative. The second shift occurred in 2003, from “GM food” to “field test.” At the same time, the GMO controversy was overcome.

Concerning stem cell research (SCR) and RM, Shineha (2016) analyzed over 7,000 news articles from the 1980s to 2013 using co-word network analysis and pointed out the peripheralization of ethical framings on SCR and RM, particularly after the appearance of human iPSCs in 2007, which increased the framings on “national promotion.” Hao and Hibino (2023) analyzed 105 news articles on brain-machine interface in Japanese and Chinese news coverage. They found that positive frames were predominant in both Japanese and Chinese news articles.

However, these previous studies have not conducted a comparative analysis between multiple fields. Further, the lack of benchmarks has caused them to fail to understand in depth the features of media discourse on each theme. This lack of knowledge is common worldwide, particularly since the 2000s (Shineha, 2016; Bauer and Gutteling, 2006; Marks et al., 2007; Nisbet and Huge, 2006; Nisbet and Lewenstein, 2002; Hibino and Nagata, 2006, 2008; Nagata et al., 2006; Shineha et al., 2008; Gilbert et al., 2019; O’Connor et al., 2012; Racine et al., 2006; Ruan et al., 2019; Stapleton and Torres Yabar, 2023; Zimmermann et al., 2019) and has prevented a comprehensive understanding of the common structure and specific contexts of each technology.

To fill this lack of knowledge, Mikihito Tanaka, one of the authors performed a preliminary study of a large dataset of Japanese and English newspaper corpus using natural language processing such as unsupervised machine learning [Mitsubishi Research Institute (MRI), 2019]. The duration, objects, and themes of the preliminary study overlapped with this study. While the results justified the propriety of the comparative data (articles and keywords) among English and Japanese newspapers and indicated the existence of ritual attitudes toward ethical issues in the Japanese press (described below), the details were blurred in a large-scale quantitative process and were yet to be declared the characteristics of ELSI in Japan.

To overcome the lack of deep understanding of the social implications of common and specific trends in fields such as RM which is reported in media discourse, this study conducted comparative long-term analysis of multiple fields by focusing on the Japanese media coverage on life science. Specifically, we concentrated on Japanese newspaper articles regarding three life sciences: GM, RM, and BS. The application of GM to medicine and industry attracted media attention from the 1980s to the early 1990s in Japan (Shineha et al., 2008). After the mid-1990s, GM food became a primary issue in media discourse on GM (Shineha et al., 2008). As such, the media discourses on GM differ from those on RM (Shineha, 2016). Regarding BS, “neuroscience” and “brain science” are often used in Japan. Particularly, “brain science” is a popular keyword, including the meanings of neuroscience and neuro-technology (Gaillard, 2018). Therefore, we used BS to include both these meanings.

These three life sciences are outstanding topics in the media, having received long-term attention (more than 20 years), which resulted in a large volume of news articles on these topics (more than 6,000) in the four major newspapers in Japan. These life sciences are expected to advance rapidly and have led to emerging technologies with broad impacts on society (e.g., genome editing in GM, medical applications of iPSCs in RM, and brain–machine interference in BS). In addition, the Japanese government has continuously invested in these three life sciences.

Considering previous studies, this study examined 30 years of media coverage and their framings on life sciences in Japan. We targeted the main periods when the three life sciences have been covered by newspapers most. First, we identified common and different points of the public discourse on the social implications of GM, RM, and BS. Second, we clarified the change of the public discourse in each field over time.

## 3 Methods

We selected four major national newspapers: *Asahi-shimbun*, *Mainichi-shimbun*, *Yomiuri-shimbun*, and *Nihonkeizai-shimbun*. They all have a large circulation, namely 5 million readers for *Asahi-shimbun*, 2 million for *Mainichi-shimbun*, 7 million for *Yommiuri-shimbun*, and 2 million for *Nihonkeizai-shimbun* in 2020. However, these newspapers have ideological differences. *Asahi-shimbun* and *Mainichi-shimbun* are considered liberal and left-leaning, while *Yomiuri-shimbun* and *Nihonkeizai-shimbun* are more conservative and right-leaning. We collected both the titles and contents of articles in these four newspapers from online database by searching for keywords. The English translations of the three Japanese keywords used for the selection of articles are “genetic modification (GM),” “regenerative medicine (RM),” and “brain-neuroscience (BS).” We excluded commercial flyers for books and seminars.

First, to determine the frequency of each theme during a specific period, we identified the time phases that correspond to the changes in the topics addressed in news articles. We conducted correspondence analysis to explore the change in topics. Correspondence analysis is a descriptive or exploratory technique used to examine how topics change over time for specific themes (Greenacre, 2016). This technique leads to data visualization in the form of contingency tables with variables (articles) as rows and categories (keywords) as columns. To this end, we used the KH coder, a software for quantitative content analysis and text mining in the Japanese language (Higuchi, 2016). This software could extract all words automatically from the sentences of articles and analyze them statistically with using correspondence analysis, which find some topical words during a specific period.

Since the number of articles was too large to code manually, we conducted a random sampling of all articles (Riffe et al., 2005). We selected articles from the relevant subgroups (phase and theme) with using a random number generator by the following stepwise rule: when there were more than 1,000 articles, we selected 100 articles; when there were from 500 to 999 articles, we selected 10% of the articles; when there were from 100 to 499 articles, we selected 50 articles; when there were <100 articles, we did not select any of them in this phase. This procedure yielded a total of 1,805 articles (GM: 650, RM:600, BS: 555) and provided the adequate minimum volume for content analysis.

Next, to describe the social implications of the three themes in Japanese newspaper articles, we modified the related frames, considering previous studies (Bauer and Gutteling, 2006; Listerman, 2010; Nisbet and Lewenstein, 2002; Hibino and Nagata, 2006, 2008; Nagata et al., 2006; Gutteling et al., 2002). Especially, we referred to five frames (utility, risk, control, fate, and morality) which Listerman (2010) proposed. Although previous studies consider frames and cultural differences in each country, these frames such as risk and morality were examined as common interests in each country (Bauer and Gaskell, 2002; Gaskell and Bauer, 2006). For modifying the frames, we conducted a mixed approach by combining deduction (top–down) with induction (bottom–up) (De Vreese, 2005). We made and modified tentative coding rules and conducted a content analysis. Then, we examined their reliability using the values of Gwet’s AC1 and Cohen’s kappa, which are described later in the comparison. These processes were conducted repeatedly, and they had the meaning of coder training.

This study aims to clarify *what* types of problems have been covered in longitudinal Japanese newspaper articles about life science topics, as well as determine *what* types of assumptions were made and *how* they were contextualized with other societal issues. While these issues are usually considered for framing analysis, this analysis does not have clear guidelines and several media studies neglected the concept due to insufficient conceptual examination (Scheufele and Tewksbury, 2007; Cacciatore et al., 2016). To avoid this mistake, we chose our conceptualization of frames by consolidating literature on news story framings. Price and Tewksbury (1997) conceptualized news story framings as an applicability effect during message processing. According to Price et al. (1997), the framing effect is embedded in text as a given message that renders the readers’ thoughts applicable to evoking particular thoughts and feelings. To this end, a study must be designed not only by investigating the accessibility of frames, such as the frequency or representation of topics but needs to also consider the applicability of frames and what types of ideas are triggered in the readers. To this end, we nominated a leader for each topic (GM, RM, RS), and after the independent organization of the frames by this person, we collated the texts of the articles by reading them together and discussed the cognitive schemes evoked by the accessible framings. These processes were recursively repeated three times. Therefore, we obtained applicable coding framings based on the reflexive processes of how the ethical issues related to emerging sciences are restricted to some typologies. Our modified coding rules of social implications are presented in Table 1.

**Table 1:** Seven frames describing social implications for the three themes.

Main-frame	Sub-frame	Explanation
1. Instrumental science		It describes technological advances as utility tools, which focus on positive expectations of technology. It covers the economic benefits of the technology and practical solutions for social issues.
2. Risky science		It describes the dangers and uncertainty of technology with paying attention to pessimistic aspects of technological advances. It covers precautionary principles and unexpected accidents.
3. Juggernaut science		It identified when no one cannot stop technological advances, which focuses on the autonomy of the progress. It provides a fatalist approach.
4. Techno-nationalism		It regards technological advance as the international competition between nations with comparing Japanese situation with foreign countries. It often emphasizes the delay of the domestic situation in technological advances.
5. Governance		It describes a call for appropriate control of technology.
	Problems and aims	It includes the morality of technology and ethical topics.
	Governance	It includes laws, ethical committees, and guidelines on technology.
	Dual use	It states that the technology can be used for different purposes from the original, especially military aims.
6. Communication matters		It emphasizes the importance of dialogs, talks, and lectures providing technological knowledge.
	Mutual communication	It describes two-way communication between scientists and ordinary people, which emphasizes the opinions of citizens, consumers, users, and patients.
	Enlightenment	It describes one-way communication from scientists to ordinary people for giving a greater understanding of the technology.
7. Trust in science		It describes trustworthiness in technological advances.
	Research integrity-system	It describes the integrity and trustworthiness of the whole research system.
	Research integrity-scientist	It focuses on the trustworthiness of individual scientists.
	Integration/reduction to science	It states that technological explanation is also applied to other fields, such as the humanities fields, which assumes that technological explanation is superior to others.

The coding was not exclusive, that is, one article could be coded under two or more frames. We assessed the reliability of the coding rule by selecting more than 10% of the observations in each phase and theme. This procedure yielded a total of 200 (GM: 70, RM:60, BS: 70) observations. The values of Gwet’s AC1 and Cohen’s kappa are shown in Table 2. Some Cohen’s kappas were relatively small but high enough to prove sufficient credibility; however, these rather smaller values were caused by the prevalence effects, for which the frames showed low frequency by both coders. However, the values of Gwet’s AC1, an indicator showing high robustness by prevalence effects (Gwet, 2014), indicated that the frames have high reliability. Considering the relative characteristics of Gwet’s AC1 and Cohen’s kappa (Vach and Gerke, 2023), The smaller gap between Gwet’s AC1 and Cohen’s kappa could be interpreted as each frame’s conceptual strictness. For example, “juggernaut science,” “techno-nationalism,” and “dual-use” frames showed the broader gaps between two indicators: These tendencies indicate that these newly introduced concepts should be interpreted cautiously. On the other hand, some frames, such as “instrumental science” and “legal governance,” showed relatively lower AC1 scores but higher kappa, which could be interpreted that these frame concepts are robust. Besides these variations, on the whole, values in both Gwen’s AC1 and Cohen’s kappa were high enough, so we concluded the reliability was sufficient for content analysis.

**Table 2:** The reliability of the frame by double coding (*n* = 200).

Main-frame	Sub-frame	Gwet AC1	Cohen’s κ
Instrumental science		0.83	0.83
Risky science		0.96	0.81
Juggernaut science		0.96	0.44
Techno-nationalism		0.95	0.59
Governance		0.83	0.79
	Problems and aims	0.94	0.68
	Legal governance	0.84	0.74
	Dual use	0.99	0.50
Communication matters	ALL	0.96	0.65
	Mutual communication	0.99	0.80
	Enlightenment	0.96	0.52
Trust in science	ALL	0.89	0.76
	Research integrity-system	0.97	0.78
	Research integrity-scientist	0.98	0.79
	Integration to science	0.94	0.68

Next, we calculated the indexes of frame diversity with using the table of percentage of frames across phases (Table 3). We used Shannon entropy as the index of frame diversity, which previous studies have applied for media analyses (McDonald and Dimmick, 2003). The calculation is as follows: H(X) = −xX P(x) log P(x), where P(x) is the proportion of each frame, x. The numbers of each frame x (or sub-frame x) were divided by the total number of articles in each phase and theme. Sub-frames were used for the diversity indexes. The higher this index of each phases and theme, the more diverse the frames. Conversely, the lower the index, the more uniform the frames.

**Table 3:** The percentage of frames across phases.

(A) Genetic modification (GM)								
Main-frame sub-frame	Phase 1 (1979–1996)	Phase 2 (1997–2002)	Phase 3 (2003–2005)	Phase 4 (2006–2009)	Phase 5 (2010–2013)	Phase 6 (2014)	Phase 7 (2015–2020)	SUM
Instrumental	56.0	23.0	34.0	44.0	39.0	50.0	54.0	42.3
Risky	18.0	24.0	15.0	14.0	21.0	26.0	29.0	20.6
Juggernaut	6.0	1.0	5.0	2.0	2.0	2.0	0.0	2.6
Techno-nationalism	4.0	3.0	2.0	4.0	6.0	10.0	9.0	5.1
Governance (ALL)	35.0	45.0	50.0	32.0	40.0	24.0	47.0	40.2
Problems and aims	3.0	5.0	3.0	3.0	1.0	0.0	6.0	3.2
Legal governance	34.0	39.0	49.0	28.0	37.0	24.0	43.0	37.2
Dual use	0.0	2.0	0.0	2.0	2.0	0.0	0.0	0.9
Others	0.0	0.0	0.0	0.0	0.0	0.0	0.0	0.0
Communication (ALL)	2.0	6.0	8.0	8.0	5.0	2.0	9.0	6.0
Mutual	1.0	3.0	4.0	1.0	1.0	0.0	2.0	1.8
Enlightenment	0.0	3.0	4.0	7.0	4.0	2.0	7.0	4.0
Others	1.0	0.0	0.0	0.0	0.0	0.0	0.0	0.2
Trust in science (ALL)	4.0	9.0	8.0	8.0	3.0	2.0	8.0	6.3
Integrity-system	2.0	2.0	6.0	1.0	1.0	0.0	2.0	2.2
Integrity-scientist	0.0	1.0	0.0	0.0	1.0	0.0	1.0	0.5
Integration	2.0	0.0	1.0	0.0	0.0	0.0	0.0	0.5
Others	0.0	6.0	2.0	7.0	1.0	2.0	5.0	3.4
Non-frame	15.0	21.0	14.0	25.0	17.0	20.0	10.0	17.2
*N*	100	100	100	100	100	50	100	650

(B) Regenerative medicine (RM)								
Main-frame sub-frame	Phase 1 (1979–1996)	Phase 2 (1997–2002)	Phase 3 (2003–2005)	Phase 4 (2006–2009)	Phase 5 (2010–2013)	Phase 6 (2014)	Phase 7 (2015–2020)	SUM
Instrumental	NA	53.0	67.0	64.0	58.0	42.0	58.0	57.0
Risky	NA	0.0	0.0	1.0	3.0	0.0	7.0	1.8
Juggernaut	NA	9.0	2.0	0.0	2.0	0.0	0.0	2.2
Techno-nationalism	NA	10.0	4.0	7.0	4.0	2.0	8.0	5.8
Governance (ALL)	NA	62.0	52.0	34.0	36.0	16.0	26.0	37.7
Problems and aims	NA	23.0	23.0	16.0	6.0	2.0	5.0	12.5
Legal governance	NA	51.0	43.0	25.0	33.0	14.0	21.0	31.2
Dual use	NA	0.0	0.0	0.0	1.0	0.0	0.0	0.2
Others	NA	0.0	0.0	0.0	0.0	0.0	0.0	0.0
Communication (ALL)	NA	3.0	2.0	3.0	5.0	6.0	2.0	3.5
Mutual	NA	0.0	0.0	0.0	1.0	0.0	0.0	0.2
Enlightenment	NA	0.0	1.0	3.0	4.0	3.0	0.0	1.8
Others	NA	3.0	1.0	0.0	0.0	4.0	2.0	1.7
Trust in science (ALL)	NA	3.0	10.0	12.0	4.0	42.0	3.0	12.3
Integrity-system	NA	0.0	5.0	5.0	0.0	20.0	3.0	5.5
Integrity-scientist	NA	0.0	5.0	9.0	4.0	33.0	2.0	8.8
Integration	NA	3.0	0.0	0.0	0.0	0.0	0.0	0.5
Others	NA	0.0	0.0	0.0	0.0	0.0	0.0	0.0
Non-frame	NA	13.0	8.0	12.0	17.0	13.0	18.0	13.5
*N*	0	100	100	100	100	100	100	600

(C) Brain-neuroscience (BS)								
Main-frame Sub-frame	Phase 1 (1979–1996)	Phase 2 (1997–2002)	Phase 3 (2003–2005)	Phase 4 (2006–2009)	Phase 5 (2010–2013)	Phase 6 (2014)	Phase 7 (2015–2020)	SUM
Instrumental	50.0	48.2	42.9	57.0	39.0	54.0	61.0	50.5
Risky	0.0	8.2	0.0	2.0	1.0	4.0	4.0	2.9
Juggernaut	6.0	4.7	5.7	3.0	3.0	2.0	2.0	3.6
Techno-nationalism	12.0	4.7	1.4	2.0	1.0	8.0	3.0	3.8
Governance (ALL)	16.0	24.7	20.0	9.0	8.0	10.0	5.0	12.6
Problems and aims	0.0	3.5	5.7	3.0	0.0	6.0	3.0	2.9
Legal governance	12.0	22.4	15.7	5.0	6.0	4.0	2.0	9.2
Dual use	4.0	0.0	0.0	2.0	2.0	2.0	1.0	1.4
Others	0.0	0.0	0.0	0.0	0.0	0.0	0.0	0.0
Communication (ALL)	8.0	0.0	0.0	4.0	6.0	0.0	1.0	2.7
Mutual	0.0	0.0	0.0	2.0	2.0	0.0	0.0	0.7
Enlightenment	6.0	0.0	0.0	1.0	5.0	0.0	1.0	1.8
Others	2.0	0.0	0.0	1.0	0.0	0.0	0.0	0.4
Trust in science (ALL)	32.0	22.4	22.9	19.0	13.0	14.0	12.0	18.4
Integrity-system	10.0	2.4	2.9	0.0	4.0	0.0	2.0	2.7
Integrity-scientist	2.0	0.0	0.0	0.0	2.0	0.0	0.0	0.5
Integration	28.0	18.8	18.6	18.0	8.0	14.0	10.0	15.5
Others	0.0	1.2	1.4	0.0	1.0	0.0	0.0	0.5
Non-frame	20.0	20.0	24.3	27.0	45.0	32.0	25.0	28.3
*N*	50	85	70	100	100	50	100	555

Finally, we conducted correspondence analysis for the coding to investigate how each theme co-occurred with other frames in one article and how it changed across phases. Using the correspondence analysis, we could visually compare between phases in each theme, themes and frames, and each theme one another in one summary figure.

## 4 Results

### 4.1 Time phases of the three life science topics

In total, we collected 37,009 (GM: 12,267, RM: 18,272, BS: 6,470) articles from 1979 to 2020 (GM: 1979–2020, RM: 1991–2020, BS: 1990–2020). The four newspapers showed a similar change in the number of articles for all three themes (the yearly change in the number of articles in each theme is presented in Supplementary Figure 1).

In this pilot study, we could not find apparent differences in the results showing topic changes in the four newspapers through correspondence analysis. Thus, we did not distinguish between the types of newspaper for the following analyses.

As a result of correspondence analysis (see the data in Supplementary Figure 2) with using a software (the KH coder), GM articles were classified into three different thematic reference periods: “fundamental and medical research,” “food application,” and “agricultural application.” “Fundamental and medical research” was the main term used during 1979–1996, and covered “clinical trials,” “experiments,” and “cells.” “Food application” was used during 1997–2002, covering “soybeans,” “label,” and “citizen.” There was controversy surrounding the safety of genetically modified foods over this period. “Agricultural application” was the main term used during 2003–2020, which covered “agriculture,” “region,” and “cultivation.” These results correspond with those of Shineha et al. (2008).

RM articles were classified into three different thematic reference periods: “ES (embryonic stem) cells,” “iPSCs,” and “STAP (stimulus-triggered acquisition of pluripotency) cells.” “ES cells” was the main term used during 1991–2005 and covered “cloning” and “fertilized eggs.” “iPSCs” was the main term used during 2006–2013 and 2015–2020 and covered “pluripotent” and “Yamanaka” (who first produced iPSCs). “STAP cells” was the main term used in 2014 and covered “misconduct” and “Obokata” (who claimed that STAP cells could be produced). STAP cells were proposed as a new method of producing stem cells, which caused considerable national interest. However, research misconduct was identified, which resulted in a national scandal (Mikami, 2018).

BS articles were classified into four different thematic reference periods: “fundamental and medical research,” “educational application,” “brain boom,” and “heath application.” “Fundamental and medical research” was the main term used during 1990–1996 and covered “cells” and “genes.” “Educational application” was the main term used during 1997–2004 and covered “learning” and “education.” “Brain boom” was the main term used during 2005–2009 and covered “Mogi Kenichiro,” who is a domestic science pundit supplying BS news commentary. There was great interest in BS in the media over this period. “Health application” was the main term used during 2010–2020, and covered “health,” “sleeping,” and “cognition.”

Thus, we chose to divide the thematic reference periods into seven phases for the analyses, as shown in Table 4.

**Table 4:** Common time phases corresponding with the change of topic.

Phase	Genetic modification (GM)	Regenerative medicine (RM)	Brain-neuroscience (BS)
1. 1979–1996	Fundamental and medical research		Fundamental and medical research
2. 1997–2002	Food application	ES cells	Educational application
3. 2003–2005	Agricultural application		
4. 2006–2009		iPS cells	Brain boom
5. 2010–2013			Heath application
6. 2014		STAP cells	
7. 2015–2020		iPS cells	

The time phases are based on the results in Supplementary Figure 2. In the Supplementary Figures 1–4, the closeness between each year shows the similarities between topics in each year. We classified each time phase according to how close the years were.

### 4.2 Media frames of the three themes

The general trends of media coverage showed that the “instrumental science” frame was dominant in all three themes (Table 3). Therefore, the positive expectation of technology occupied the press discourse surrounding these technologies in life science.

We also found different trends for the three themes. Regarding GM, the frequency of the “risky science” frame was higher and the frequency of the “trust in science” frame was lower than those of the other frames (Table 3A). This indicated that the negative arguments surrounding GM mainly focused on food safety and environmental risks, but not on the trustworthiness of this technology. Regarding RM, the frequency of the “problems and aims” frame was higher, especially in phases 2 and 3 (1997–2005), and the frequency of the “trust in science” frame was higher in phase 6 (2014) than that of the other frames (Table 3B). The former finding indicated that many ethical committees on ES cells were held in this phase. This finding indicated that research misconduct related to STAP cells was dominant in 2014. Regarding BS, the frequency of the “integration/reduction to science” frame was higher than that of the other frames (Table 3C), which indicated that explanations from BS could often be applied to the humanities field. In addition, the frequency of “non-frame” (i.e., the article could not be coded under any of our frames) was the second highest in BS and higher than in other themes. This result suggests that a large portion of the articles on brain technology were “straight news” and did not mention any social implications.

The pattern of the sub-frames varied for “trust in science.” “Integration/reduction to science” showed different patterns from “research integrity-system” and “research integrity-scientist.” “Integration/reduction to science” had a 10–28% frequency in BS articles, while “research integrity-scientist” had only a 0–2% frequency (Table 3C). “Research integrity-system” and “research integrity-scientist” had frequencies of 33 and 20%, respectively, in phase 6 of RM, but “integration/reduction to science” did not appear in that phase of RM (Table 3B).

### 4.3 Diversity of frames

**Figure 1:**
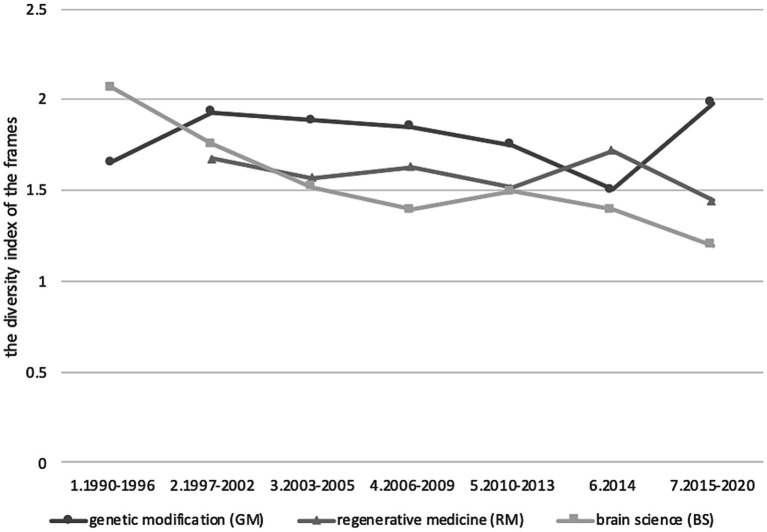
The diversity of frames for the three themes. The X axis shows time phases and the Y axis the diversity index of the frame for each theme.

The time changes in the diversity index of the frames significantly differed among the three themes (Wilcoxon signed-rank test, all *p* < 0.05, GM vs. BS: V = 28, GM vs. RM: V = 21, BS vs. RM: V = 21, Figure 1). The indexes of diversity were not correlated among these themes across the time phases (Spearman’s rank correlation test, all *p* > 0.05, GM vs. BS: S = 64, GM vs. RM: S = 52, BS vs. RM: S = 26).

The diversity index of GM articles showed a somewhat cyclical pattern for the time phases, that is, the GM diversity index increased from phase 5 (2010–2013) or phase 6 (2014) to phase 7 (2015–2020). The diversity index of RM articles was fixed at more than 1.5 and the diversity index of BS articles constantly decreased over the time phases.

### 4.4 Results of correspondence analyses

**Figure 2:**
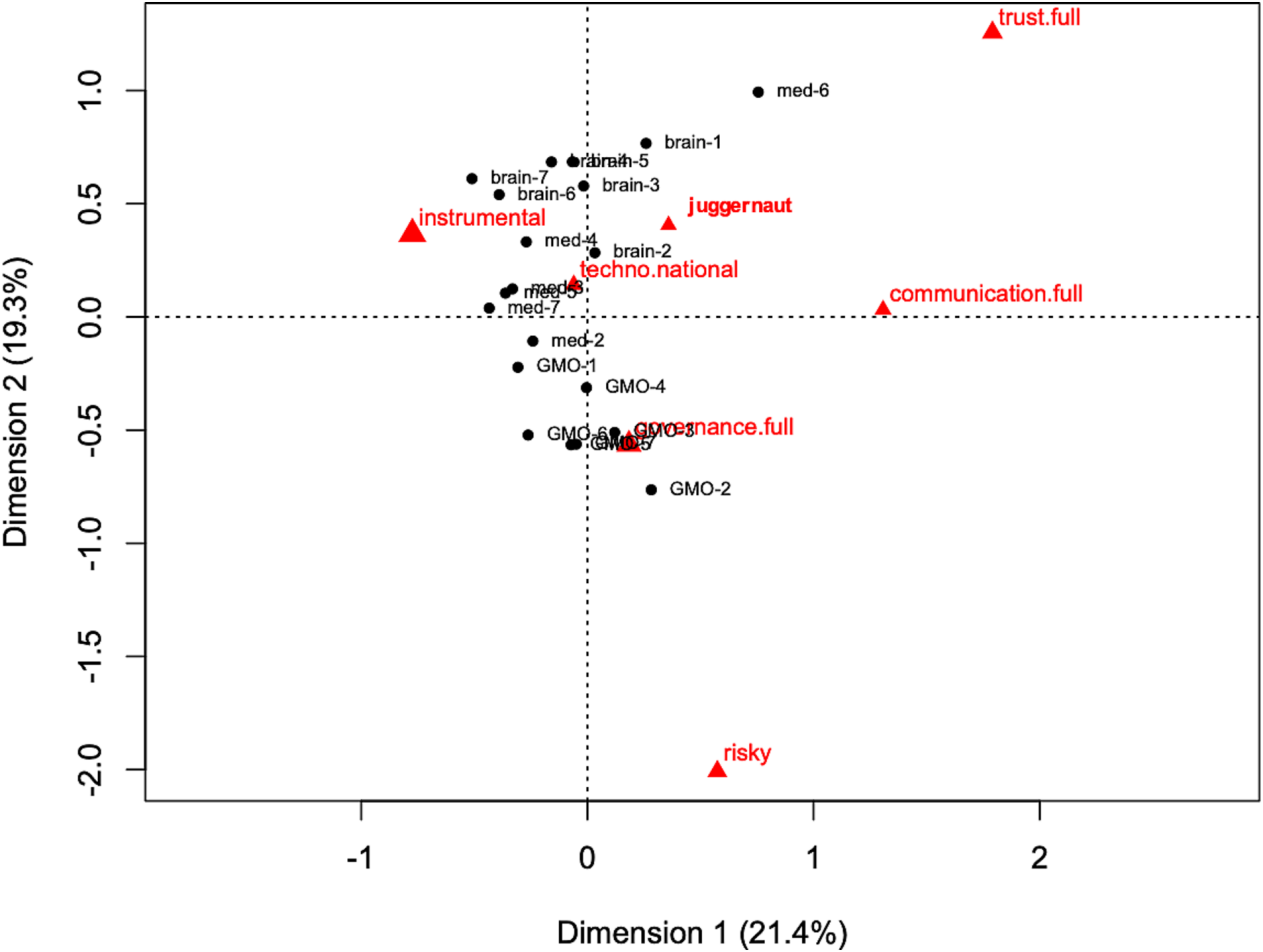
Correspondence of all themes with the frames for the seven phases. Notes: The circles indicate the average values of each theme (GMO, genetic modification; med, regenerative medicine; brain, brain-neuroscience) and each phase (each number indicated the time phase), and the triangles indicate the frames. Each circular dot shows the average position of the themes for each phase. The percentage of each axis shows the contributing rate of each score (i.e., data from multiway contingency tables), which indicates how the axis of word associations could explain the variation between scores.

The correspondence analyses revealed the relative positions of the main frames in each theme and phase (Figure 2 and Supplementary Figure 3). Each circular dot shows the average of values from each article of each theme and phase. The percentage of each axis shows the contributing rate of each sample score, which indicates how the axis of frames could explain the variation of samples. These data were interpreted by the configuration of relative locations between themes or phases (for details, see Hibino and Nagata, 2006). For example, in Figure 2 “med-7” and “med-6” are relatively located away from each other, however “med-7” and “med-5” are close to each other. This indicated that regarding frames, “med-7” was similar to “med-5,” and different from “med-6.” The closer to the origin (0, 0) of the coordinate, it had the more similar with other samples (e.g., “thechno.national” in Figure 2). Conversely, the closer to the margins of the coordinate, it had more different from other samples (e.g., “trust.full” in Figure 2).

The sample scores were generally plotted around “instrumental science” (Figure 2), which indicates that positive opinions of technology were dominant in media coverage. In particular, BS articles were more associated with this frame. Both the “juggernaut” and the “techno-nationalism” frames were plotted near the center on the coordinate. The results showed that these frames co-occurred with other frames. Meanwhile, “risky science,” “trust-in science,” and “communication” were plotted near the margins of the coordinate, as these frames occurred solely in one article. Although both BS and RM became slightly closer to “instrumental science” as the phase progressed, the change across phases was not generally coordinated for these themes.

Our results also show that the three themes had the following different patterns. Of the three themes, GM articles were associated more with “risky science” and “governance” than with other themes (Figure 2). This indicates that GM articles had more negative opinions than the other themes. In the single GM analysis, “instrumental science” was closely associated with “techno-nationalism” (Supplementary Figure 3). The dominant frames in GM articles changed from “instrumental science” to “governance”; in other words, phases 1, 5, and 6 (1971–1996, 2010–2014) were associated with the former frame and phases 2, 3, 4, and 7 (1997–2009, 2015–2020) were associated with the latter one.

Regarding RM, “instrumental science,” “governance,” “techno-nationalism,” and “juggernaut” tended to co-occur together in one article (Supplementary Figure 3). Although phase 6 (2014) of RM was plotted around “trust-in science,” other phases of RM were generally plotted in the same surroundings of these three frames. This indicates that the type of the frames was largely constant across the phases of RM, except for 2014, when research misconduct related to STAP cells was reported.

Regarding BS, “instrumental science” was constantly dominant across the phases. The “trust in science” frame was associated with “governance” (ethical) in the single BS analysis (Supplementary Figure 3).

## 5 Discussion

Our analyses found common and different points concerning how the Japanese media had covered GM, RM, and BS. Regarding the common points, the “instrumental science” frame was the most frequent among the three themes, which indicated that positive arguments were generally dominant. This is consistent with previous Japanese studies (Shineha, 2016; Hibino and Nagata, 2006) and international studies (Nisbet and Lewenstein, 2002; Gilbert et al., 2019; O’Connor et al., 2012; Ruan et al., 2019; Gutteling et al., 2002; Kamenova and Caulfield, 2015) of the media discourse on life science. This suggests that the frames covered by the majority of articles on social implications are fixed to a particularly positive one.

Although the majority of articles are written in positive tone with an “instrumental science” frame, there are articles with a combination of the seven frames, particularly in GM and RM cases. Regarding the different points between three themes, GM covered more diverse frames than the other themes. In particular, there were phases when “governance” was higher than instrumental (phases 2, 3, and 5: 1997–2005, 2010–2013), which contrasted with other frames, in which “instrumental science” was the highest in any phase. Furthermore, “risky science” had the highest frequency in GM compared with the other themes and was unique in that it was mentioned alone. The diversity index of GM was also higher in the 5/7 periods (Figure 1), which indicated that GM covered the most diverse issues, including negative and positive views of technology in media coverage.

Regarding articles on RM, the issue of regulatory ethics followed events of clinical application and industrialization. Phases 2, 3, 4, 5, and 7 (1997–2013, 2015–2020) were associated with the “instrumental,” “juggernaut,” “techno-nationalism,” and “governance” frames (Supplementary Figure 3). Typical RM articles emphasized the usefulness of RM, described that no one could stop technological advances, or that Japan should lead the international competition, and also pointed out that the discussion of appropriate regulation and ethical issues was essential. In this context, the concept of governance was regarded as a requirement for promoting RM. Previous research has argued that RM tended to be optimistically reported in the UK, US, and Canada (Kamenova and Caulfield, 2015) and also in Japan (Shineha, 2016). Our results supported this trend.

Regarding BS articles, we identified an emphasis on its instrumental aspects throughout all phases. A sense of instrumentality in BS is enhanced through the expectations toward the advancement of technology. Most prominently, medical research with BS creates high expectations for a wide range of applications, such as a cure for Alzheimer’s disease or dementia, or preventive medicine. In addition, BS knowledge is considered to be applicable not only to medicine but also to non-science fields, such as education and marketing.

Although each theme is adjacent to one another, our results suggested the public discourse of each theme do not overlap but are independent of one another. The frame diversity for each phase differed between the three themes and were not correlated among them (Figure 1). Our correspondence analyses showed that the phases tended to be plotted by grouping those of the same theme on the coordinate (Figure 2). These results support the independence of themes. Most content within an article did not refer to other themes.

This independence might reflect the different histories among the three themes. In GM articles, the relatively high frequency of “governance” frames in each phase was related to policy movements. In detail, the approach to “governance” in the correspondence analysis (phases 2 and 3: 1997–2005) was strongly related to the regulation of GM food labeling (phases 2 and 3: 1997–2005). The phase of relatively high “governance” frames (the top three are phases 2, 3, and 7) was strongly related to the establishment of guidelines for genome editing technology (phase 7: 2015–2020), which coincided with policy changes. In addition, these results are consistent with previous studies (Shineha et al., 2008). This response for genome editing contrasted with those for other themes, possibly because GM had a past history with governance affairs (i.e., the safety of GM foods), while RM and BS did not. This revival of the governance frame might be explained by the issue-attention cycle model (Downs, 1972). According to the issue-attention cycle, themes that attract hype progress rapidly in the aftermath of that hype. In addition, the emergence of genome editing technology (phase 7: 2015–2020) was linked to the rise in frame diversity, suggesting that novel technologies would lead to frame diversification in GM articles.

Our results generally coincided with those of other countries (especially, for European countries) and past cases (Bauer and Gutteling, 2006; Gilbert et al., 2019; O’Connor et al., 2012; Zimmermann et al., 2019; Gutteling et al., 2002; Eyck and Williment, 2003; Dobmeier et al., 2023; Fischer and Hess, 2022), despite differences in factors such as cultural backgrounds. This implies that past issues were important factors determining the present discourse in media coverage.

We also found some backlash against the three technologies. GM articles often picked up the negative opinions of consumers, especially in phases 2 when the safety of GM foods was debated. RM articles sometimes mentioned negative opinions on the application to fertilized eggs, such as the fear about the operation of life, especially in phases 2 and 3. Although there were very limited cases, some BS articles were cautious about the widespread use of BS. In some cases, the media took up the worry that the boom would foster public misperceptions of BS. Terms such as “the myth of brain-neuroscience” and “pseudo (brain) science” showed up in articles. Simultaneously, the role and responsibility of experts were discussed.

In addition, the arguments regarding philosophical and ethical problems overall did not evolve or deepen over time. Especially in RM articles, ethical discussions were not substantive but superficial and ritualistic, such as “It’s going to spark an ethical debate,” “We need to have an ethical debate,” and “The ethical aspect will be an issue.” Although the reason why ethical discussions in Japanese newspapers have been limited is beyond the scope of this study, a roundtable discussion conducted by Japanese science journalists based on the preliminary research [Mitsubishi Research Institute (MRI), 2019] presents several possibilities for the reasons for the ritual nature of the Japanese ethical debate, which was also found in this study (Pandemic ELSI, 2024). Firstly, Japanese science reporting has developed alongside national policy science, such as nuclear power and space development. For this reason, Japanese science reporting is used to provide knowledge commentary to help the public understanding of science or to criticize policy, but it is not used to question the nature of science itself fundamentally. Related to the first point, framing discussions about ethics also leads to questioning the responsibility of each individual citizen. It seems that framing the news in this way deviates from the critical approach to power that is the norm in Japanese journalism, and this leads to a lack of focus on ethical issues. What is more, it seems that this is not just a problem for science journalists, but also for experts in the humanities and social sciences in Japan. According to the roundtable discussion, when science journalists ask experts in the humanities and social sciences for their comments on ethical issues like this, they often express a resigned attitude that “there are some things that cannot be disputed in the face of scientific progress.” This is the very “juggernaut science” that we found in this study. In other words, juggernaut science is a way of thinking about science that is prevalent not only among science journalists but also among Japanese ethicists and others. The mechanisms by which Japanese science-related reporting avoids ethical issues should be examined further in the future, but it is thought that these historical and cultural aspects are having an impact.

Nevertheless, it must be noted that not all the ethical issues were ignored in the Japanese press. “Trust in science,” particularly on research ethics issues such as fabrication, strongly attracted media attention in Phase 6. In BS articles, the variety of issues discussed in philosophy and ethics communities (Bublitz and Merkel, 2014; Council of Europe, 2021; Ienca, 2021; Ienca and Andorno, 2017; Ienca and Haselager, 2016; Lavazza, 2018; OECD, 2019; Presidential Commission for the Study of Bioethical Issues, 2014, 2015; Shen, 2013) was absent in the media coverage. It is evident that there is a huge gap in interests between the current discussions on social implications in academia and in the media.

Interestingly, the current survey showed that public attitudes toward genome-edited food are not negative compared to those toward GM food (Kato-Nitta et al., 2019). As our study found, media coverage on GM articles including genome-edited foods has a variety of framings, compared to the other two fields (RM or BS). This reflects the accumulation of controversies regarding GMO (particularly GM foods) in 1990s. One reason for the change from GMO to genome-edited food in public attitudes seems to be the accumulation of communications, dialogs, and controversies with various framings. The lessons learned from the GMO case will be useful for the consideration of other cases.

## 6 Conclusion

Our study examined how the social implications of three life sciences were represented in Japanese media coverage by analyzing relevant articles in four daily newspapers. The common trends of media coverage showed that the “instrumental science” frame was dominant, indicating that positive opinions of life science dominated media coverage. Our results also showed that the time change of frames varied by theme, and the diversity index of the frames differed significantly among the three themes. This finding implied that the background of time changes differed from theme to theme, and that there was little common background or influence on each other.

Regarding GM, the articles were associated with “risky science” and “governance,” indicating that the press covered more negative arguments than for the other themes. Regarding RM, “instrumental science,” “techno-nationalism,” “juggernaut science,” and “governance” co-occurred more often in one article, indicating that governance was aimed at promoting the development of technology. Regarding BS, the “instrumental science” frame was constantly dominant in the research period and dominated the other themes. In summary, GM had relatively diverse frames, including risks, while both RM and BS were limited to the “instrumental science” frame, thus preceding the expectation of utilization.

Our research adds to the basic knowledge of how public discourses of emerging life science have featured in media coverage. However, future research needs to clarify why each similar theme is independent of the other themes in the framing of media coverage.

Our study confirmed that the media tends to report positive expectations of life science in Japan. Therefore, this indicates a huge gap between professional discussions of ethics community and Japanese media coverage. An urgent task is to bridge this gap, for which we must recognize the need for ethics communication and science communication. These practices can contribute to better understanding and deliberating the social implications of emerging life science. Our findings on the framing of ELSI in mass media discourse in Japan will provide basic information on this gap.

## Data Availability

The raw data supporting the conclusions of this article will be made available by the authors, without undue reservation.
