# Supplementary material for: Comparative analysis of media coverage concerning the social implications on three life sciences in Japan during 1991–2020
Source: Front Sociol. 2025 Mar 11;10:1523795. doi: 10.3389/fsoc.2025.1523795 (PMC11933884; doi:10.3389/fsoc.2025.1523795)
Supplement: Supplementary file 1 [file Table_1.DOCX]

Supplement 1.

the yearly change of the number of articles in each theme

Supplement 2.

These figures show the results of correspondence analyses of each theme with keywords (Japanese) for each year. The plot near the origin was expand by three times in all these figures. Each square showed the average of samples for each year. The percentage of each axis showed the contributing rate of each sample score (i.e., each year), which indicates how the axis of keywords could explain the variation of samples. These data were interpreted by the configuration of relative locations between keywords or years (for details, see [29]). The simple Euclidean distance was available for evaluating the strength of the relationship between each year. We set the criterion of the times for 100-150 analyzed words.

Correspondence analysis of GM in Yomiuri-shimbun


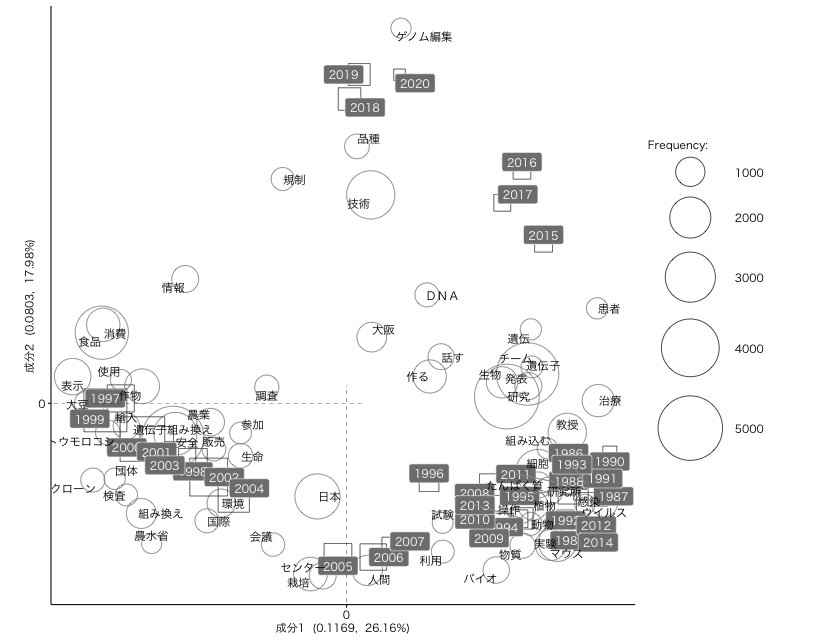


Top 60 keywords filtered by chi-square value from 105 words that appeared more than 450 times.

Correspondence analysis of GM in Aasahi-shimbun


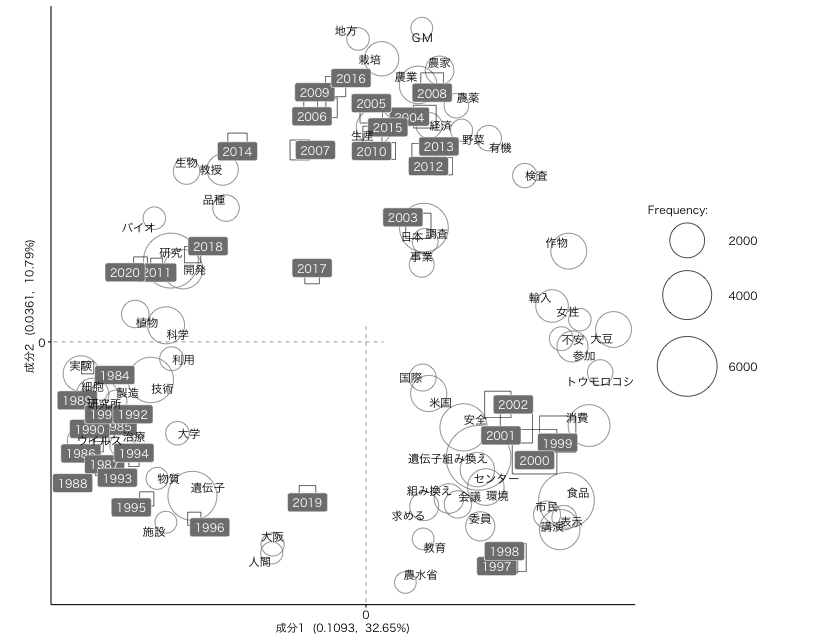


Top 60 keywords filtered by chi-square value from 118 words that appeared more than 750 times.

Correspondence analysis of GM in Mainichi-shimbun


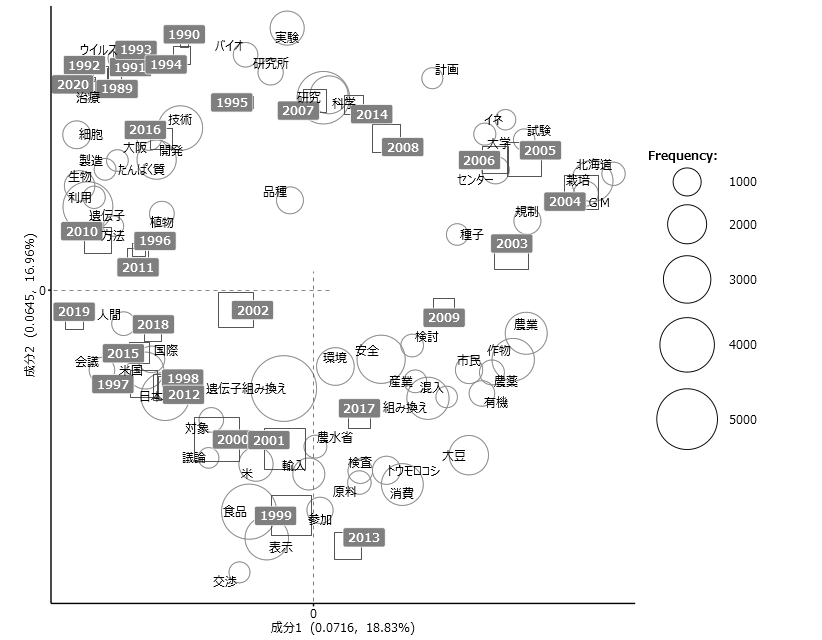


Top 60 keywords filtered by chi-square value from 135 words that appeared more than 500 times.

Correspondence analysis of GM in Nikkei-shimbun


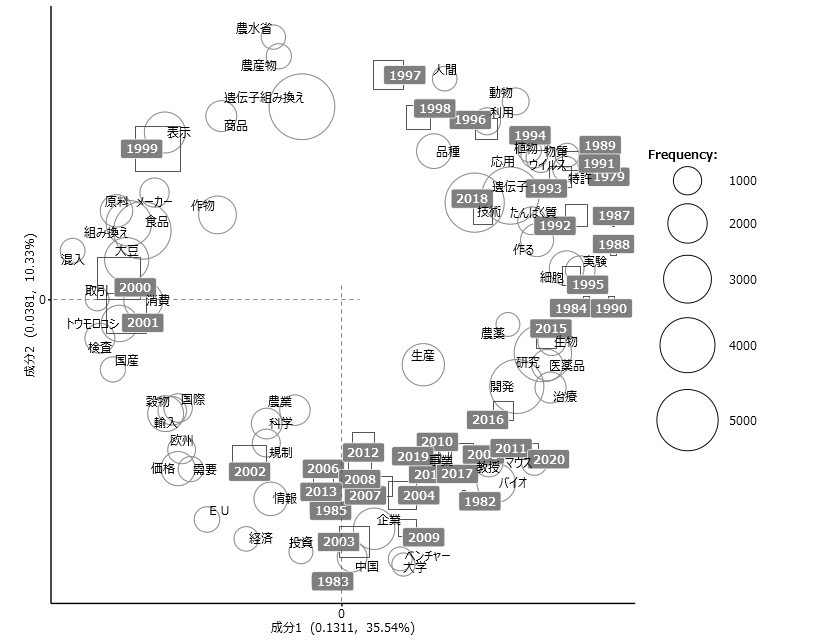


Top 60 keywords filtered by chi-square value from 107 words that appeared more than 650 times.

Correspondence analysis of RM in Yomiuri-shimbun


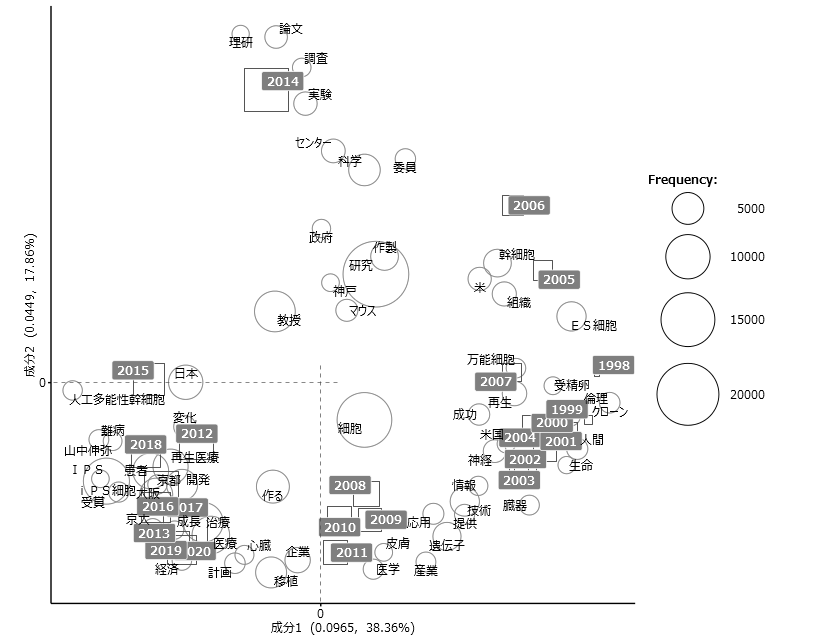


Top 60 keywords filtered by chi-square value from 105 words that appeared more than 1300 times.

Correspondence analysis of RM in Asahi-shimbun


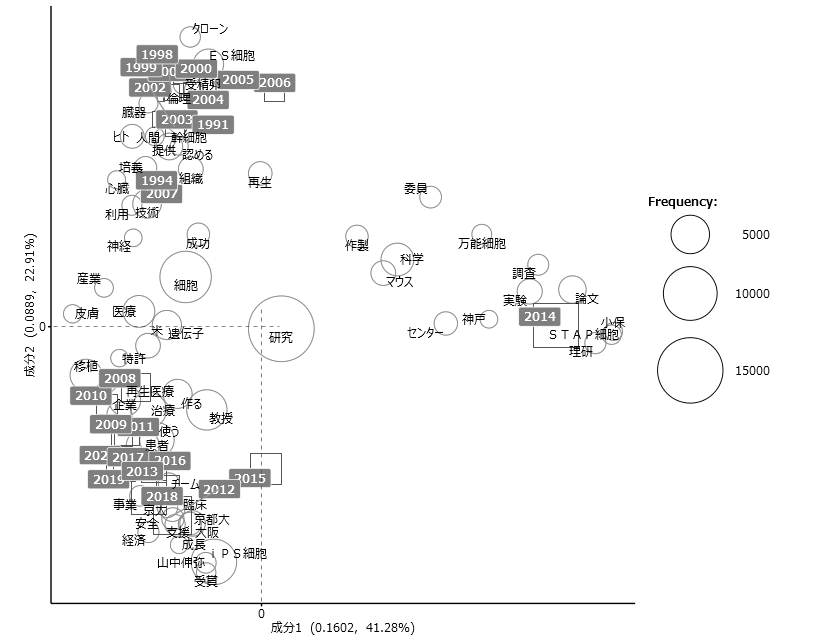


Top 60 keywords filtered by chi-square value from 106 words that appeared more than 900 times.

Correspondence analysis of RM in Mainichi-shimbun


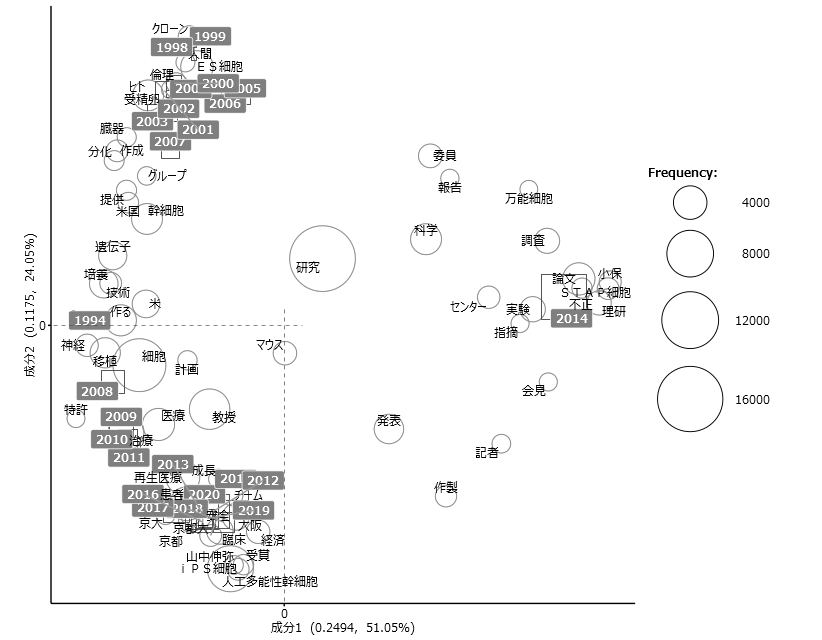


Top 60 keywords filtered by chi-square value from 107 words that appeared more than 1000 times.

Correspondence analysis of RM in Nikkei-shimbun


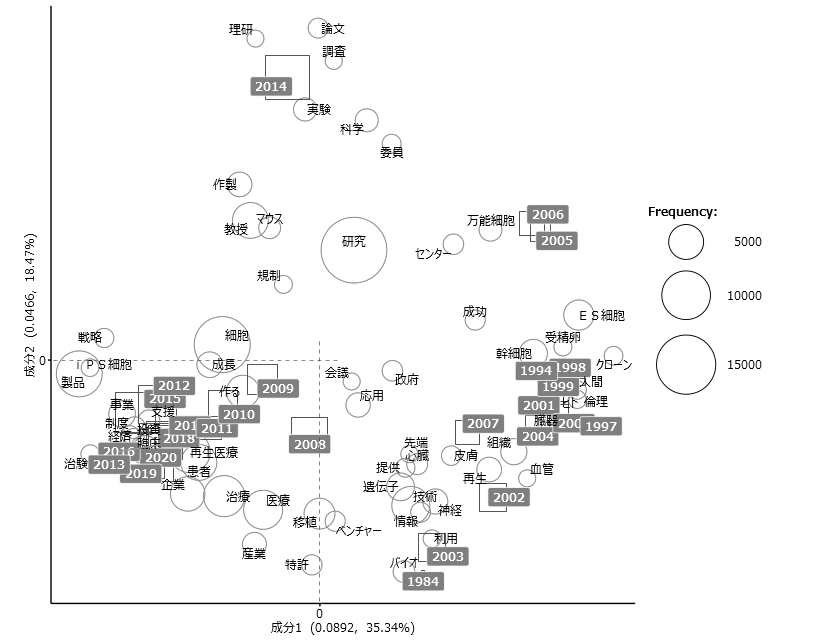


Top 60 keywords filtered by chi-square value from 117 words that appeared more than 1050 times.

Correspondence analysis of BS in Yomiuri-shimbun


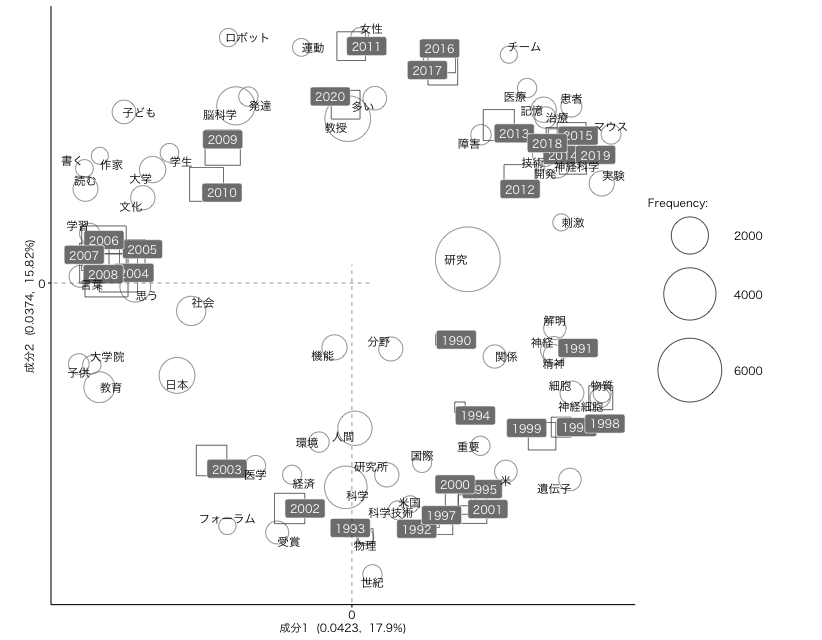


Top 60 keywords filtered by chi-square value from 119 words that appeared more than 400 times.

Correspondence analysis of BS in Aasahi-shimbun


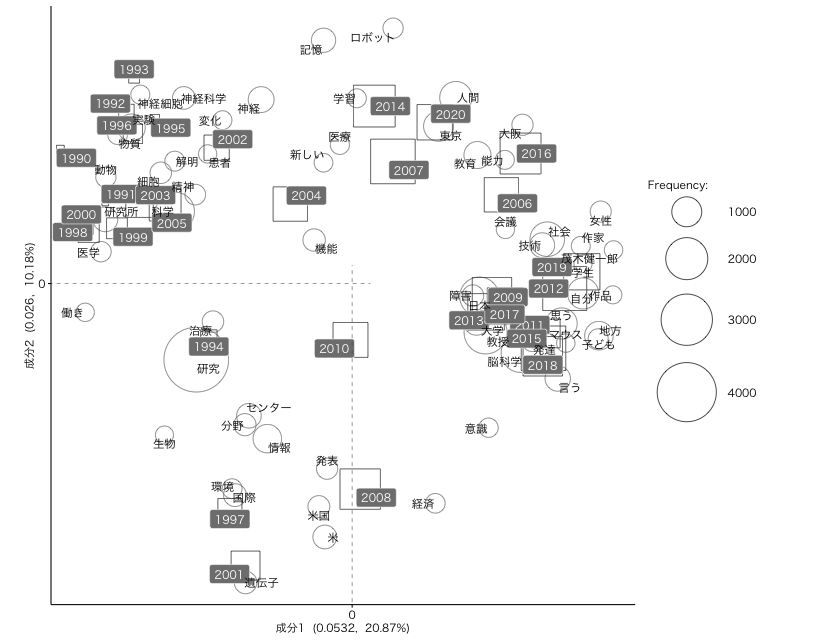


Top 60 keywords filtered by chi-square value from 110 words that appeared more than 350 times.

Correspondence analysis of BS in Mainichi-shimbun


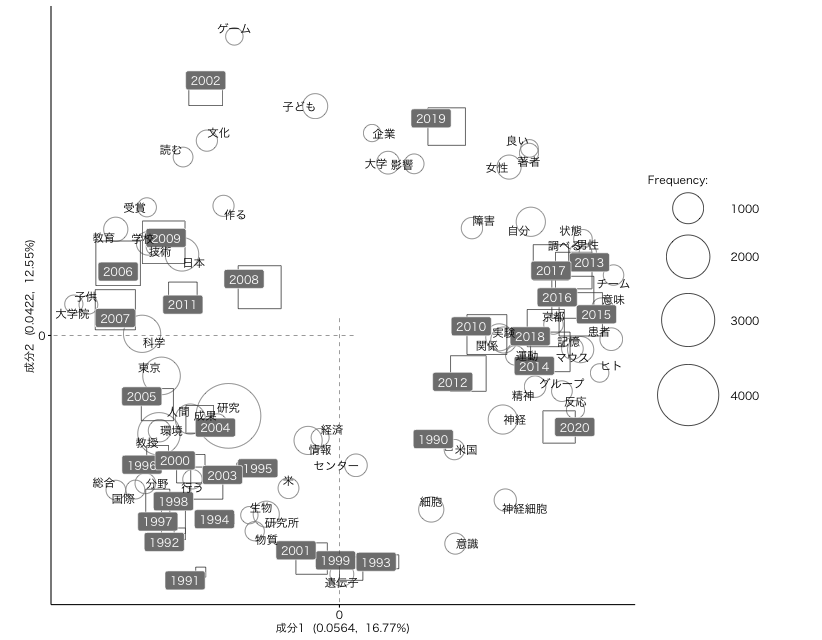


Top 60 keywords filtered by chi-square value from 123 words that appeared more than 300 times.

Correspondence analysis of BS in Nikkei-shimbun


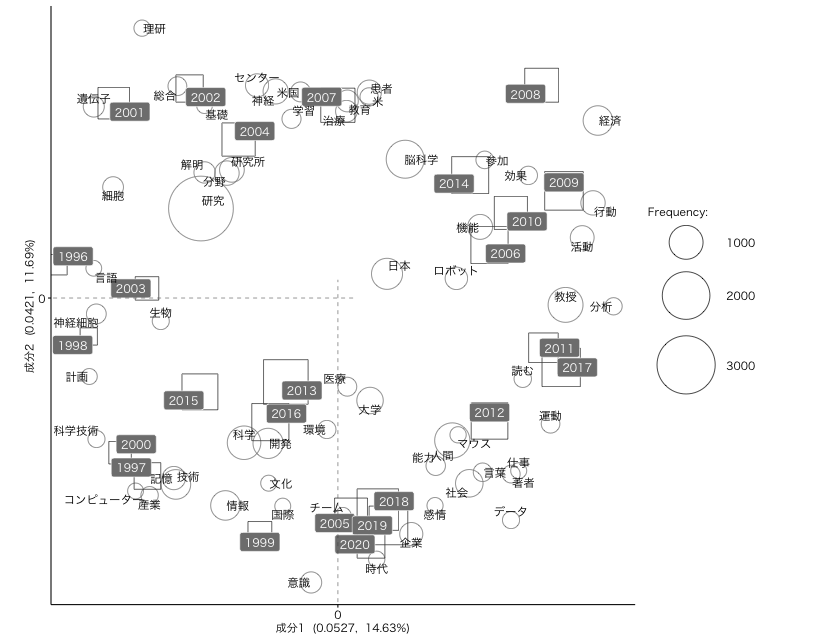


Top 60 keywords filtered by chi-square value from 124 words that appeared more than 200 times.

Supplement 3.

Correspondence analyses of each theme with frames for the seven phases. (a) genetic modification (GM), (b) regenerative medicine (RM), (c) brain-neuroscience (BS). The circles indicated the average values of each theme and each phase, and the triangles indicated the frames. Results of correspondence analysis of sub-frame were shown in Supplement 4.

Supplement 4.

Correspondence analyses of each theme with sub-frames for the seven phases. The circles indicated the average values of each theme and each phase, and the triangles indicated the frames. The frequency of sub-frame, which were more than 1 % (in GM and BS) or 5 % (in RM), were included in these analyses.

GM

RM

BS
